# Posterior cruciate ligament recruitment affects antero-posterior translation during flexion gap distraction in total knee replacement: An intraoperative study involving 50 patients

**DOI:** 10.3109/17453674.2010.501743

**Published:** 2010-07-16

**Authors:** Petra Heesterbeek, Noël Keijsers, Wilco Jacobs, Nico Verdonschot, Ate Wymenga

**Affiliations:** ^1^Department of Research, Development and Education, Sint Maartenskliniek, Nijmegen; ^2^Orthopaedic Research Laboratory, Radboud University Nijmegen Medical Centre, Nijmegen; ^3^Laboratory for Biomedical Engineering, University of Twente, Enschede; ^4^Department of Orthopaedics, Sint Maartenskliniek, NijmegenThe Netherlands

## Abstract

**Background and purpose:**

Because of the oblique orientation of the posterior cruciate ligament (PCL), flexion gap distraction could lead to anterior movement of the tibia, which would influence the tibiofemoral contact point. This would affect the kinematics of the TKR. We assessed the flexion gap parameters when the knee is distracted during implantation of a PCL-retaining TKR. Furthermore, the effects of PCL elevation (steep or flat) and collateral ligament releases on the flexion gap parameters were determined.

**Methods:**

During a ligament-guided TKR procedure in 50 knees, the flexion gap was distracted with a double-spring tensor with 200N after the tibia had been cut. The flexion gap height, anterior tibial translation, and femoral rotation were measured intraoperatively using a CT-free navigation system.

**Results:**

During flexion gap distraction, the greatest displacement was seen in anterior-posterior direction. Mean ratio between increase in gap height and tibial translation was 1 to 1.9, and was highest for knees with a steep PCL (1 to 2.3). Knees with a flat PCL and knees with a ligament release had a larger increase in PCL elevation when the gap was distracted.

**Interpretation:**

When the PCL is tensioned, every extra mm that the flexion gap is distracted can be expected to move the tibia anteriorly by at least 1.7 mm (flat PCL), or more if there is a steep PCL. This changes the tibiofemoral contact point, which may have consequences for polyethylene wear.

## Introduction

Total knee replacement (TKR) with retention of the posterior cruciate ligament (PCL) is a successful procedure with excellent long-term results (Rand et al. 2003). However, to realize the potential benefits of a PCL-retaining design, proper balancing and a good surgical technique are essential (Tanzer et al. 2002, Most et al. 2003). Inadequate correction of the soft tissue imbalance in both the sagittal and coronal planes can lead to non-physiological kinematics and an adverse tibiofemoral contact point (Banks et al. 2003, Yoshiya et al. 2005) and is an important factor related to early TKR failure (Pagnano et al. 1998, Sharkey et al. 2002). In a TKR with a tibiofemoral contact point that is located too anteriorly on the tibia, the maximum flexion is limited by posterior impingement of the tibial insert against the back of the femur (Bellemans et al. 2002). In addition, the efficiency of the extensor mechanism is reduced and the knee can be unstable. On the other hand, when the tibiofemoral contact point is too posterior, there is high pressure on the posterior part of the polyethylene insert and flexion is also limited because of a tight PCL (Swany and Scott 1993). Several studies have shown the importance of balancing the PCL in a PCL-retaining TKR, but have also indicated that an optimal PCL balancing may be difficult to achieve (Nozaki et al. 2002, Straw et al. 2003).

During surgery, the PCL is balanced by distraction of the flexion gap through spacers, spreaders, and tensors. The flexion gap is the 3-dimensional space defined by the bony surfaces of the femur and tibia and by the surrounding soft tissue structures of the knee (i.e. the capsule and the (collateral) ligaments) and exists only under distraction (Mihalko et al. 2000). When the flexion gap is distracted, repositioning of the femur and tibia is often observed; the tibia translates anteriorly with regard to the femur (Christen et al. 2007) and the femur may also rotate because of the constraints of the ligaments. When soft-tissue structures around the knee (e.g. the collateral ligaments) are released during ligament balancing, the behavior of the flexion gap during flexion gap distraction may change. Similarly to the gap distraction for ligament balancing, the thickness of the polyethylene insert will also affect femur-tibia repositioning. Mahoney et al. (1994) showed that a thicker insert substantially increased the PCL strain. However, they did not quantify the effect of the increased strain on femur-tibia repositioning.

In this study, we hypothesized that the so-called “dynamics” of the flexion gap is dependent on the orientation of the PCL since this obliquely oriented structure is the strongest ligament crossing the knee, and is known to be the major restraining structure in 90° of flexion (Covey and Sapega 1994, Harner et al. 1999, Amis et al. 2003). Christen et al. (2007) reported a high variation among patients in the relation between flexion gap increase and translation of the tibia. Based on that study, we also hypothesized that variations in individual PCL orientation lead to inter-patient variations in flexion gap parameters and that ligament releases may also play a role. To our knowledge, a direct relation between PCL orientation and the flexion gap dynamics has not been reported in the literature.

The first goal of our study was to determine the quantitative changes in the parameters for the flexion gap (gap height, tibial antero-posterior translation, medio-lateral displacement, and femoral rotation) when the knee is distracted with a tensor intraoperatively during TKR, and to determine whether knees with ligament releases have different dynamics. The second goal was to determine the orientation of the PCL in the gap and to assess whether flexion gap kinematics can be predicted from PCL orientation. If this was confirmed, the surgeon could more predictably decide for each patient the specific consequences of the magnitude of the change in flexion gap (e.g. by increasing the polyethylene insert thickness) on 3D repositioning by considering the orientation of the PCL.

## Patients and methods

In this prospective study, 50 knees in 50 patients (mean age 63 (44–84) years, 32 females) undergoing primary total knee replacement were included. All patients were on a waiting list for a posterior cruciate retaining primary total knee replacement (CR-TKR) for osteoarthritis with a fixed varus or valgus deformity of less than 10°. Inclusion criteria were primary osteoarthritis and intact PCL as assessed by macroscopic inspection by the surgeon. Exclusion criteria were traumatic osteoarthritis, a BMI of > 30, and ipsilateral total hip replacement. The study was approved by the local Institutional Review Board (CMO-nr: 2004/180), and all patients provided written informed consent for participation in the study.

### Surgical technique and measurements

A total condylar fixed-bearing PCL-retaining TKR, the ligament-guided balanSys system (Mathys Ltd., Bettlach, Switzerland) was implanted. The surgery was performed using a CT-free navigation system (Surgetics; Praxim, La Tronche, France). The standard surgery protocol for navigated surgery was followed with preservation of the PCL insertion by a bony island through a so-called “V-cut”. The hip center was calculated after rotating the leg to determine the leg axis (e.g. point of least movement) and the center of the ankle was calculated using the medial and lateral malleoli, which were digitized by a pointer with reflective markers. The femur axis was defined by a line through the hip center and the intercondylar notch (also digitized). The tibia axis was defined as a line through the intercondylar spine on the tibia plateau and the center of the ankle. The medial and lateral epicondyles were also digitized. The arthritic bony surfaces of the tibia and femur were digitized with the pointer; the surfaces of the joint were “scanned” and samples were automatically taken by the computer. The shape of a standardized bone geometry was morphed onto these surface points (Bonemorphing).

**Figure 1.**
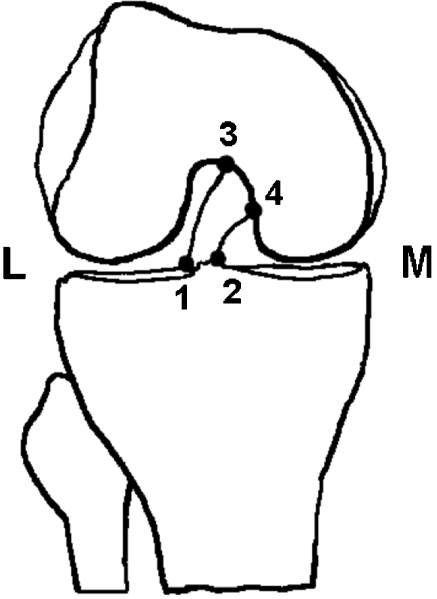
Additional points selected with pointer as input to determine the location of the femoral and tibial PCL insertion sites.

In this study, the navigation system was also used as a measurement device to indicate the orientation of the PCL. Since this measurement module was not included in the regular surgical protocol, the most medial and most lateral points of the attachment sites of the PCL on the tibia and femur were indicated with the pointer in the wound (Figure 1). Afterwards, the midpoints between the femoral and tibial points of the attachment sites were calculated and connected, and defined as the PCL. After the tibia was cut with a posterior slope of 7°, the knee was flexed to approximately 90° to perform the measurements with the calibrated tensor (balanSys; Mathys Ltd.). An adapted tensor was used for the measurements; the blades were polished and lubricated in order to enable free antero-posterior movement during distraction. The flexion gap was distracted sequentially with 100 and 200 N. The force was applied perpendicular to the blades of the tensor (and perpendicular to the tibial slope). Movements of the reference frames of the tibia and femur as a consequence of the tension forces applied were registered by the camera of the navigation system. Movements of 0.5 mm or 0.5° could be detected by the camera, and the total accuracy of the system was 1 mm or 1°. The flexion gap dynamics and PCL elevation outcome parameters were determined and quantified as described below.

### Flexion gap dynamics

**Figure 2.**
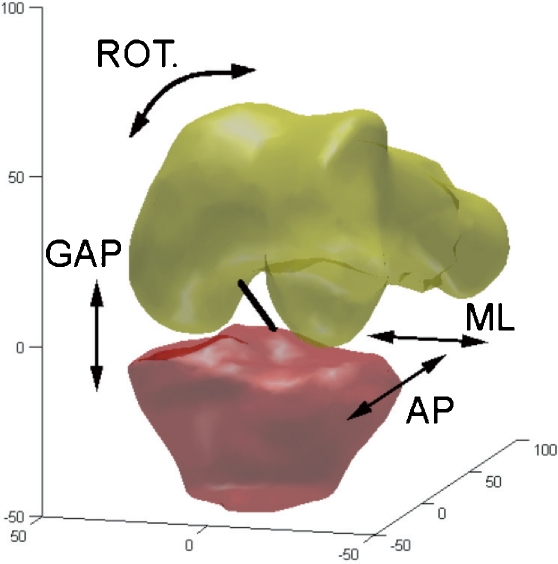
3D view of the knee with the parameters of interest: gap height (GAP), anterior-posterior translation (AP), medio-lateral displacement (ML), and rotation of the femur (ROT.).

The dynamics of the flexion gap were described by the change in gap height, anterior translation, medial-lateral displacement, and femoral rotation when the flexion gap was distracted with 100 and 200 N. Measurements of an untensioned flexion gap (0 N applied with the tensor) were not considered reliable measurements because there is no “filling” between the femur and tibia after the tibial osteotomy, and their relative position could be arbitrary. With the application of 100 N (and 200 N), the flexion gap is definitely distracted and 200 N was considered a safe amount of maximum distracting force, and therefore, the difference between 100 N and 200 N was chosen to assess the flexion gap dynamics. The origins of the femur (intercondylar notch of the femur) and tibia (intercondylar spine of the tibia) were used to calculate the flexion gap parameters in 3 dimensions. The increase in gap height was defined as the translation of the origin of the femur in the direction of the proximal-distal axis through the tibia (Figure 2). The increase in anterior tibial translation was calculated from the same point but in the direction of the anterior-posterior tibial axis.

Medial-lateral displacement was defined as movement of the origin of the femur with regard to the medio-lateral axis of the tibia. Finally, the femoral external or internal rotation of the femur relative to the tibia during gap distraction was determined by the difference in orientation of the transepicondylar axis relative to the tibia between 100 N and 200 N of flexion gap distraction. The transepicondylar axis was defined as the line between the medial and lateral epicondyles as indicated by the surgeon. The knee angle was determined by calculating the angle between the tibia axis and the femur axis in order to control for changes in PCL elevation induced by changes in knee angle. To minimize noise, we took a “window” with a width of 5 consecutive points, centered on the maximum value of all parameters of interest, and averaged these 5 points (i.e. the maximum and 2 points before and 2 points after the maximum). This mean value was then used to describe the flexion gap parameters.

### PCL elevation

**Figure 3.**
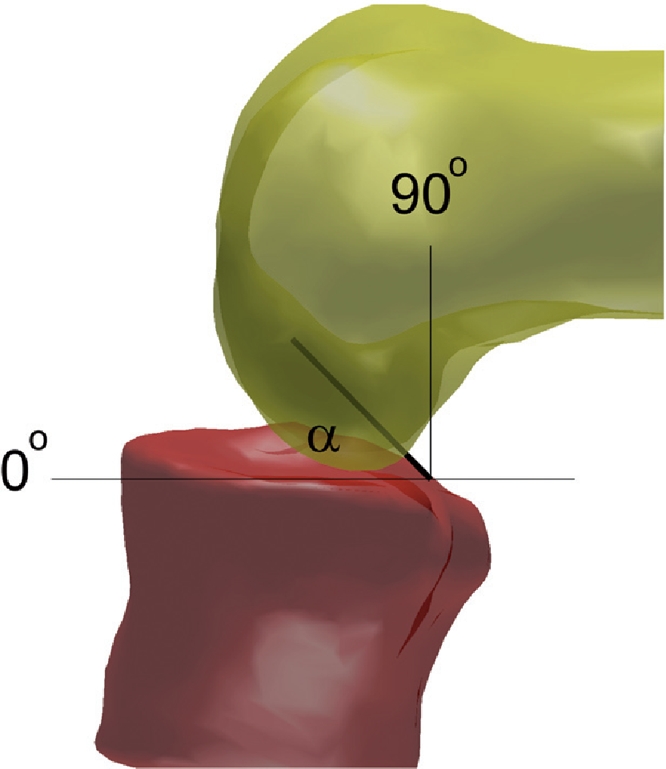
Schematic illustration of PCL elevation, which is the angle (α) between the (cut) tibial surface (not illustrated) and the calculated PCL (black line).

To determine whether the flexion gap dynamics can be explained by the orientation of the PCL, the exact locations of the PCL insertion sites were identified. With the custom-written software program, the centers (Cf and Ct) of the most medially and laterally localized attachments of the PCL on the femur and tibia were calculated. The PCL orientation was described and defined as PCL elevation (PCLe). PCL elevation was calculated as the angle between the “calculated” PCL (line between Cf and Ct) and the transverse plane of the tibia (Figure 3).

### Releases

The surgeon may need to perform a ligament release in extension to align the leg in cases of varus or valgus deformity. Releases were performed according to the principle “tightest structure first”, and as many releases were performed as necessary to create neutral leg alignment (mechanical axes of femur and tibia are 180°). The release of a structure in extension with a stabilizing effect in both extension and flexion may affect the measurements in flexion (Krackow and Mihalko 1999a, b). In our study, a ligament release is defined as the release of a structure with a major stabilizing effect in flexion. A medial stabilizer in flexion is the (anterior part of the) medial collateral ligament and lateral stabilizers in flexion are the popliteus tendon complex and the capsule of the posterolateral corner (Whiteside 2002). If one of these structures was released during surgery, then that knee was categorized as having a “release”. When none of these structures or another structure was released (i.e. stabilizers in extension), knees were categorized as “no release”. For the sake of simplicity, medial and lateral releases were considered as one group. All releases performed were recorded.

### Analysis and statistics

Custom-written software in MatLAB (version 7.0; The Mathworks Inc., Natick, MA) was used to calculate flexion gap dynamics and PCL elevation. To identify the relation between flexion gap height increase and anterior tibial translation, the ratio between anterior translation and flexion gap height increase was determined. To investigate the orientation of the PCL, the elevation angle of the PCL (PCLe) was calculated for 100 N as well as the change in elevation angle (ΔPCLe) between 100 N and 200 N.

Theoretically, if AP movement between the tibia and femur is guided by the PCL as we hypothesized, an elevation below 45° (flat PCL) would have little effect on the AP tibiofemoral motion; a more steeply-oriented PCL (above 45°) would have more effect on AP movement. Thus, we introduced these two categories (flat or steep) for PCL elevation in our dataset.

A factorial ANOVA (between groups) was used to analyze the data. A release and the orientation of the PCL were included as independent variables, and their effect on the flexion gap dynamics, on the gap-anterior translation ratio, and on the change in PCL elevation (all three dependent variables) was investigated. The results are presented as mean (SD), with an α-level of 0.05 being considered significant.

## Results

### Flexion gap dynamics

**Figure 4.**
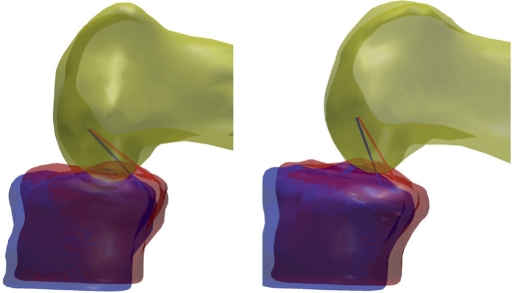
3D illustration of 2 knees in sagittal view (no fibula). The situation with 100 N of tension is shown in red, and that with 200 N is shown in blue. The amounts of gap height increase and anterior tibial translation are visible and different for the knee with a flat PCL (left) and the knee with a steep PCL (right). Note the increase in PCL elevation after 200 N has been applied (blue PCL).

Figure 4 illustrates the flexion gap dynamics between 100 and 200 N as observed in this study. Interestingly, the largest amount of displacement was not seen in the proximal-distal (gap) direction, but in the anterior-posterior direction. The least amount of movement was seen in the medial-lateral direction (Table 1). Rotation of the femur remained limited to an average of 0.63° (SD 1.4) exorotation for all groups together, ranging from 2.1° endorotation to 5.3° exorotation. Mean ratio between gap height increase and anterior tibial translation was 1 to 1.9 (0.59); on average, for each mm of increase in gap height, the tibia moved forward 1.9 mm.

**Table 1.** Mean (SD) gap increase (Gap), medio-lateral displacement (ML), anterior tibial translation (AP), and femoral rotation (Rot) after tension increased from 100 N to 200 N

		PCLe < 45°	PCLe ≥ 45° **^a^**
Release –	Gap (mm)	2.5 (0.6)	1.8 (1.5)
	ML (mm)	–0.06 (1.6)	–0.2 (1.4)
	AP (mm)	4.2 (1.5)	3.8 (2.6)
	Rot (°)	0.9 (1.4)	–0.01 (1.1)
	n	27	6
Release + **^b^**	Gap (mm)	3.2 (0.9)	2.0 (0.7)
	ML (mm)	–1.1 (2.7)	–0.7 (1.7)
	AP (mm)	5.5 (1.9)	4.2 (1.1)
	Rot (°)	0.2 (1.5)	0.7 (1.1)
	n	10	7

**^a^** Statistically significant effect of PCLe on gap (p = 0.001); no effect of PCLe on ML, AP, or Rot.

**^b^** Effect of release on gap not statistically signifiant (p = 0.09); no effect of PCLe on ML, AP, or Rot.

### PCL elevation

Mean PCL elevation was 41° (8.3) when the flexion gap was distracted with 100 N. Mean ΔPCLe between 100 and 200 N was 9.9° (3.9).

When the flexion gap was tensioned with 100 N, the mean angle between femur and tibia was 97° (4.0). When 200 N of tension was applied, the mean knee angle increased to 99° (4.4). Although this increase of 1.5° was small, almost every patient showed an increase and this increase was therefore statistically significant (p < 0.001).

### Effect of PCL elevation

**Figure 5.**
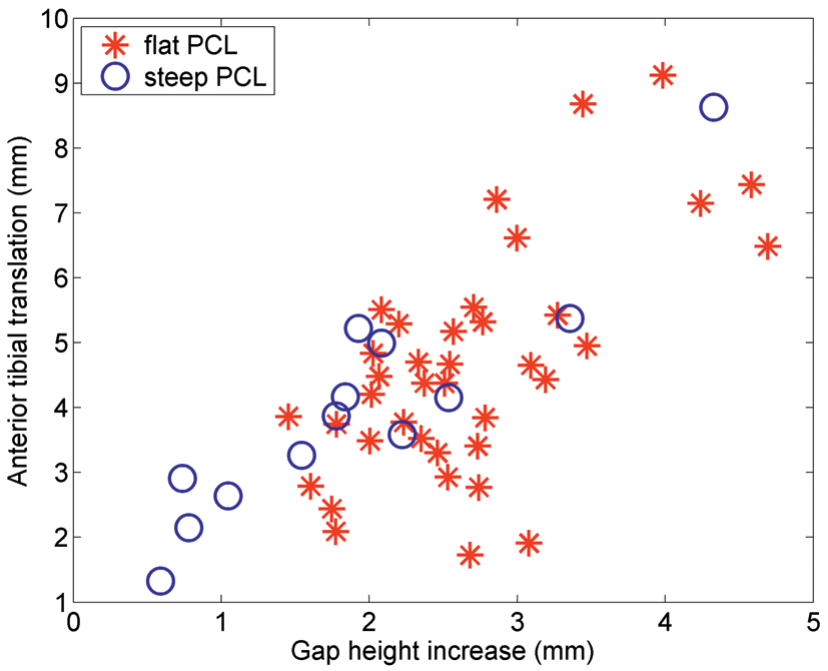
Scatter plot of change in gap height against change in anterior tibial translation for knees with a flat or a steep PCL after the flexion gap tension has increased from 100 N to 200 N. Each entry represents one patient (n = 50).

Affirming our hypothesis, we did indeed find an effect of PCL elevation on the ratio between gap height increase and anterior tibial translation (p = 0.002) (Figures 4 and 5). In knees with a steep PCL, AP translation increased more for each mm of increase in gap height (gap/AP ratio was 1:2.3 (0.63)) compared to knees with a flat PCL (gap/AP ratio was 1:1.7 (0.50)) (Table 2). Furthermore, PCL elevation influenced the absolute increase in gap height (p = 0.001); patients with a flat PCL (PCLe < 45°) had a larger gap height increase than patients with a steep PCL. Hence, a steeper PCL seemed to resist the distraction force more effectively. In addition, PCL elevation increased more in knees with a flat PCL than in knees with a steep PCL when the flexion gap was distracted (p = 0.02) (Table 3). On the other hand, PCL elevation had no statistically significant effect on ML displacement, AP translation, or femoral rotation.

**Table 2.** Mean (SD) AP/gap ratio

		PCLe < 45°	PCLe ≥ 45° **^a^**
Release −	AP/gap	1.7 (0.5)	2.5 (0.8)
	n	27	6
Release + **^b^**	AP/gap	1.7 (0.4)	2.2 (0.4)
	n	10	7

**^a^** Statistically significant effect of PCLe on AP/gap ratio (p = 0.002).

**^b^** No effect of release on AP/gap ratio.

**Table 3.** Mean (SD) ΔPCLe

		PCLe < 45°	PCLe ≥ 45° **^a^**
Release –	ΔPCLe (°)	9.1 (2.3)	7.1 (4.8)
	n	27	6
Release + **^b^**	ΔPCLe (°)	13.6 (4.1)	10.2 (4.9)
	n	10	7

**^a^** Statistically significant effect of PCLe on ΔPCLe (p = 0.021); flat PCLs (PCLe < 45°) had greater ΔPCL.

**^b^** Significant effect of release on ΔPCLe (p = 0.002); knees with a release had a greater increase in PCL elevation.

### Effect of releases

**Figure 6.**
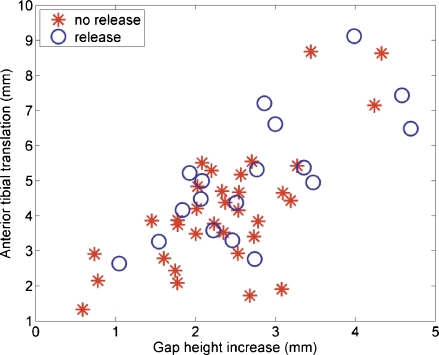
Scatter plot of change in gap height against change in anterior tibial translation for knees with or without a release after the flexion gap tension has increased from 100 N to 200 N. Each entry represents one patient (n = 50).

Knees with a release had a greater increase in PCL elevation as the distracting force increased from 100 N to 200 N (p = 0.002). PCL elevation increased the most for knees with a flat PCL and a release (Table 3). There was an effect of release on gap height increase, although not statistically significantly so (p = 0.09). There was no significant difference in mean ratio of gap height increase and anterior tibial translation between knees with a release and knees without (Figure 6, Table 2). In addition, a release did not have any statistically significant effect on AP translation, ML displacement, or femoral rotation. No interaction effects between PCL elevation and releases were found for any of the outcome parameters.

## Discussion

### Flexion gap dynamics

We found a strong association between flexion gap height increase and anterior tibial translation. For every single mm of increase in gap height, induced by either a tensor or by an additional 1-mm polyethylene insert, the tibia translates forward by 1.9 mm on average. It is remarkable that distraction in proximal-distal direction led to this substantial displacement in antero-posterior direction. The absolute amount of gap height increase and anterior tibial translation varied between patients, which might be explained by inter-individual differences in behavior and orientation of the PCL. In addition, some knees apparently have tighter ligamentous or capsular structures whereas other patients may have knees that are naturally more compliant.

To date, only one other study has investigated the quantitative relationship between flexion gap distraction and tibial translation (Christen et al. 2007). That study found a mean relation of 1:1.25 between gap height and forward tibial translation after flexion gap distraction with a monoblock tensor. This lower amount of tibial translation and hence a less strong relation may be explained by the difference in tensors used. With a monoblock tensor, the most restraining structure determines the amount of translation whereas with a bi-compartimental tensor, as used in the present study, the translation may be less restrained and therefore higher. Furthermore, the measurements in our study were conducted 3-dimensionally using a navigation system, whereas Christen et al. used the less accurate scale on the tensor.

We found only limited femoral rotation during flexion gap distraction. The collateral ligamentous structures play a prominent role in the resulting amount of rotation when a tensor is used, and the PCL is only a secondary restraint for femoral rotations because of its small moment arm (Amis et al. 2003). The restraining structures in flexion were apparently balanced; i.e. there was no sizeable difference between medial and lateral laxity.

### PCL elevation

The mean PCL elevation was 41° (26–65) in a tensioned situation (100 N) at 97° of flexion. In the literature we found an elevation angle of 24° at 90° of knee flexion, measured as the angle between the longitudinal axis of the tibia and the PCL with MRI in young adults (Komatsu et al. 2005). To evaluate similar angles, the value for PCLe in the young adult study was subtracted from 90°, resulting in 66°. This is slightly higher than the value for PCL elevation in our study (41°). A more extreme value for PCL elevation was reported by Nakagawa et al. (2004): the PCL approached the vertical when knee flexion increased from 60° to 120°. We could not confirm that. Another study measured the angles between the different bundles of the PCL and the tibial plateau and reported that as knee flexion increased from 0° to 120°, the angle of the anterolateral bundle increased from 37° to 65° and that of the posteromedial bundle increased from 47° to 60° (Zaffagnini et al. 2004). Although the elevation angle in our study was measured at a more or less constant knee angle and no distinction was made between the anterolateral and posteromedial bundles of the PCL, the reported values were within the same range; in our study, the PCL elevation increased from 41° to 51° when the flexion gap was distracted from 100 N to 200 N. We do not feel that our results were influenced by the increase in knee angle that occurred during distraction. Although this increase was statistically significant, the effects due to this 1.5° reduction of flexion angle on the relative position of the measuring points were considered negligible.

### The effect of PCL elevation

Our hypothesis that the dynamics of the flexion gap depend on the orientation of the PCL was confirmed. Distraction of the knee with a low PCL elevation angle (i.e. a flat PCL) resulted in greater increase in gap height than distraction of a knee with a steep PCL. This result was to be expected; when the gap between the tibia and femur was increased, the increase in PCL tension resulted in the tibia pivoting around the femoral insertion of the PCL. Thus, the greatest increase in gap height will occur when the PCL is flat.

Taking this finding into consideration, the surgeon should be aware of the strong effect of PCL elevation on the relationship between gap height increase and tibial translation. A flat PCL will lead to a mean anterior tibial translation of 1.7 mm when the gap is distracted 1 mm. A knee with a steep PCL will even translate 2.3 mm on average with 1 mm of gap distraction. If the PCL were the only structure that was tensioned with distraction of the flexion gap, then at 100 N it would be oriented vertically. This is not the case, and therefore other structures such as the collateral ligaments must have restrained the translation. From an anatomical point of view, the collateral structures are the only structures that could have had a restraining function on vertical movements. For obvious reasons, in this in vivo study we could not test what would have happened to the orientation of the PCL when the collateral structures were resected.

In addition, the PCL elevation angle at 100 N influenced the increase in PCL elevation angle between 100 N and 200 N (ΔPCLe). This seems logical: a flat PCL can be recruited more easily whereas knees with a steep PCL might already be tensioned at 100 N so that a further increase in PCL elevation angle would be hard to achieve. However, steep PCLs are not necessarily recruited more than flat PCLs. Furthermore, the situation during implantation of a TKR is quite unnatural: we investigated the PCL elevation after the tibia was cut.

### Effect of releases

Knees with a collateral ligament release tended to have a greater flexion gap height increase. An increased gap height would be the logical consequence of a collateral ligament release, since the loosening of this structure would reduce the constraining function of the collateral ligament. A release apparently did not influence the relative dynamics of the flexion gap: knees with or without a release showed the same relation between gap height increase and anterior tibial translation.

We found that flexion gap distraction of knees with a collateral ligament release led to a greater increase in PCL elevation, thus confirming the restraining function of the collateral ligaments during flexion gap distraction. A release reduces this restraining function of the ligaments; thus, the PCL will be recruited more and will guide the AP movement of the tibia more effectively, which would result in a greater change in PCL elevation angle.

### Clinical implications

Our findings have implications for clinical practice. A 2-mm additional polyethylene insert not only produces an increase in gap height but can also result in anterior tibial displacement that cannot be ignored. With this 2 mm of extra polyethylene, the tibia could translate anteriorly between 3.4 and 4.6 mm in 90° of flexion, depending on the elevation of the PCL. However, in practice most surgeons use an insert with a conforming dish with a posterior lip that prevents tibial translation. However, since anterior tibial displacement is restrained, the forces on the posterior part of the polyethylene will increase and may eventually cause PE damage. In this situation, the PCL would be tensioned too tightly. The flexion gap would be too tight, with a tibiofemoral contact point that is too posterior, causing limited flexion and pain. During surgery, this can be recognized as “open-book kinematics” with tibial tray lift-off in flexion during trial implantation (Ritter et al. 1988, Swany and Scott 1993, Nozaki et al. 2002). On the other hand, should the flexion gap not be sufficiently distracted, i.e. an undersized PE, then the PCL may be too loose. This will result in flexion laxity with increased anteroposterior laxity, and a contact point that is too anterior—resulting in limited flexion because of impingement of the femur on the back of the tibial plateau. The effects described are valid for 90° of flexion. Thus, with normal gait the effects would be limited. In the young and active patient, however, flexion instability is an important issue and should therefore be taken into account.

We analyzed the effect of an increase in gap distraction force in an already tensioned flexion gap. In surgical terms, what happens when a thicker polyethylene insert is used? Although our study has given some insight into flexion gap dynamics, we still do not have an ideal recipe for the surgical technique. We found an average of 119° of 1-year postoperative range of motion and an average AP laxity of 3 mm in 90° of flexion in a cohort of 50 cruciate retaining TKAs all implanted with 100 N of distraction force in flexion (unpublished data). It is unclear whether 100 N is suitable for every patient. The distraction force should probably be individualized for each patient. Also, further insight is needed into how the surgeon can technically control the contact point between tibia and femur, especially on the constrained medial side since this is in our view the key to good flexion, low polyethylene stresses, and a stable knee in flexion.
